# Performance of BioFire array or QuickVue influenza A + B test versus a validation qPCR assay for detection of influenza A during a volunteer A/California/2009/H1N1 challenge study

**DOI:** 10.1186/s12985-021-01516-0

**Published:** 2021-02-25

**Authors:** David R. McIlwain, Han Chen, Maria Apkarian, Melton Affrime, Bonnie Bock, Kenneth Kim, Nilanjan Mukherjee, Garry P. Nolan, Monica M. McNeal

**Affiliations:** 1 Department of Pathology, Stanford University School of Medicine, Stanford, CA USA; 2 WCCT Global, Cypress, CA USA; 3 ARK Clinical Research, Long Beach, CA USA; 4 Department of Pediatrics, University of Cincinnati College of Medicine, Division of Infectious Diseases, Cincinnati Children’s Hospital Medical Center, Cincinnati, OH USA

**Keywords:** Influenza, H1N1, Volunteer influenza challenge study, Biofire film array, Rapid influenza diagnostic test, RIDT, QPCR

## Abstract

**Background:**

Influenza places a significant burden on global health and economics. Individual case management and public health efforts to mitigate the spread of influenza are both strongly impacted by our ability to accurately and efficiently detect influenza viruses in clinical samples. Therefore, it is important to understand the performance characteristics of available assays to detect influenza in a variety of settings. We provide the first report of relative performance between two products marketed to streamline detection of influenza virus in the context of a highly controlled volunteer influenza challenge study.

**Methods:**

Nasopharyngeal swab samples were collected during a controlled A/California/2009/H1N1 influenza challenge study and analyzed for detection of virus shedding using a validated qRT-PCR (qPCR) assay, a sample-to-answer qRT-PCR device (BioMerieux BioFire FilmArray RP), and an immunoassay based rapid test kit (Quidel QuickVue Influenza A + B Test).

**Results:**

Relative to qPCR, the sensitivity and specificity of the BioFire assay was 72.1% [63.7–79.5%, 95% confidence interval (CI)] and 93.5% (89.3–96.4%, 95% CI) respectively. For the QuickVue rapid test the sensitivity was 8.5% (4.8–13.7%, 95% CI) and specificity was 99.2% (95.6–100%, 95% CI).

**Conclusion:**

Relative to qPCR, the BioFire assay had superior performance compared to rapid test in the context of a controlled influenza challenge study.

**Supplementary Information:**

The online version contains supplementary material available at 10.1186/s12985-021-01516-0.

## Background

Influenza remains a major global health concern with significant morbidity and mortality from seasonal infections and poses the potential for catastrophic pandemics [1]. In addition to the human cost, influenza infection also results in a tremendous economic burden with more than 20 million days of lost annual productivity and an estimated $11.2 billion in annual direct or indirect costs in the United States alone [2].

Distinguishing influenza infection from other acute respiratory conditions based on symptoms alone is difficult [3], but timely and accurate detection of influenza infection is a key component of both global disease surveillance monitoring, individual clinical case management, and clinical studies. A gold-standard for definitive diagnosis of influenza infections is a quantitative real-time polymerase chain reaction (qPCR) assay where the copies of viral nucleic acid in clinical samples are quantified and compared to a standard curve produced using the same strain [4, 5]. However, standard qPCR approaches are time-consuming and require trained operators in a laboratory setting [5]. Other options for more rapid diagnosis are designed to overcome some of these limitations, sample-to-answer PCR-based systems and point-of-care immunoassay based rapid influenza diagnostic tests (RIDT) [6]. Sample-to-answer qPCR-based systems typically simplify workflow by automating sample nucleic acid extraction, using reagent cassettes that include pre-loaded positive and negative controls, and by generating easy to interpret outcome reports in about 1 h. RIDTs on the other hand are primarily lateral flow immunoassays that rely on antigen detection in biological samples with colorimetric readouts. Such tests provide results in 15 min or less, are simple to perform, and are generally at a lower cost than PCR-based methods [7]. However, the performance characteristics of each assay should always be a major consideration when choosing an influenza test.

Controlled human infection studies where healthy volunteers are challenged with influenza virus are important tools for the evaluation of novel influenza therapeutics [8–10]. Such studies also provide a rare opportunity to examine the performance characteristics of influenza diagnostics in a highly controlled setting. While multiple studies have reported the relative performance of different influenza detection methods [11], such assays are rarely (if ever) compared using samples obtained from a cohort of individuals all exposed to the same dose of a genetically identical virus at a known point in time. Samples obtained in a volunteer A/California/2009/H1N1 influenza challenge study were separately compared to test outcomes of a validated qPCR assay versus a sample-to-answer qPCR device (BioMerieux BioFire FilmArray RP) and a lateral flow immunoassay based RIDT (Quidel QuickVue Influenza A + B Test). Understanding the relative performance of these methods in this context has important implications for the design of future influenza challenge studies.

## Methods

### Influenza challenge study design and sample collection

During a volunteer influenza challenge study (ClinicalTrials.gov Identifier: NCT02918006) [12], 143 volunteers with low levels of pre-existing antibodies (HAI titers of less than 1:20) were challenged intranasally with an identical dose of A/California/2009/H1N1 challenge virus stock (a post-hoc qPCR assay determined each dose to be between 5.50 × 10^5^ to 3.5 × 10^6^ TCID_50_ units [12]. This same dose of this same virus stock was sufficient to result in virus shedding in 67% of healthy volunteers in a prior dose-ranging study [13]. The presence of virus shedding was strongly correlated with the appearance of volunteer reported symptoms. Please see Liebowitz et al. [12] for additional details on symptomology, inclusion criteria, demographics of volunteers, and other information. Nasopharyngeal (NP) swabs (Quidel, San Diego, CA) were collected up to twice daily by inserting the swab into the nasopharynx and turning before placement in a supplied tube containing universal virus transport medium. NP swab specimens were used for the detection of virus shedding by a validated qPCR assay (qPCR) throughout the study. Starting on day 4 post virus challenge, matched NP swabs were collected from individual volunteers at each time point and the presence of virus was evaluated using a qPCR assay and either the BioMerieux BioFire FilmArray RP (BioFire) or Quidel QuickVue Influenza A + B Test (rapid test).

### Quantitative real-time PCR (qPCR) assay and qualification

A real-time PCR (qPCR) method was adapted from a method developed at the US Centers for Disease Control and Prevention (CDC) and validated at the Laboratory for Specialized Clinical Studies at Cincinnati Children’s Hospital Medical center. The assay detected and quantified shedding of Influenza A/California/04/2009 H1N1 virus in clinical samples. Nucleic acid extraction of 140µL of the NP swab samples was carried out by use of the Qiagen QIAamp Viral RNA Mini Kit (Qiagen, Hilden, Germany). Primers and probes (Biosearch Technologies, Inc, Novato, CA) targeting the HA gene of the pandemic (pdmH1) influenza A (H1N1) 2009 virus were used (Additional file 1: Table S1). To evaluate the quality of the NP swab samples, a separate PCR reaction was performed to detect the Human RNPase P gene. Detection of this gene confirms that the swab sample is of sufficient quality that cell-associated virus can be detected and quantified and acts as an internal control for any possible PCR inhibitors in the swab sample. A one-step quantitative RT-probe Hydrolysis kit, Ambion AgPath-ID™ One-Step kit (Thermo Fisher, Waltham, MA) was used in the PCR reaction following the manufacturer’s instructions. The final concentration of primers was 0.8 µM and 0.2 µM for the probe. 5µL of the extracted material was used in each reaction. PCR conditions using an Applied Biosystems ABI 7500 PCR system (Thermo Fisher, Waltham, MA) were as follows: 50.0 °C for 30 min; 95.0 °C for 10 min; 45 cycles of 95 °C, 15 s followed by 55 °C for 34 s.

To develop a standard curve for quantitation, the HA gene sequence was obtained from GenBank (KU933485.1) for A/California/07/2009. A forward primer at positions 1–25 (ATGAAGGCAATACTAGTAGTTCTGC) with a 5′ T7 promoter and a reverse primer at positions 1702–1673 (TTAAATACATATTCTACACTGTAGAGACCC) were used to generate a transcript of 1702 base pairs in a one-step RT PCR reaction. The product was run on a 1% gel and the band was purified with the Zymoclean Gel DNA Recovery Kit (Zymo Research, Irvine, CA). A Megascript T7 transcription kit (Thermo Fisher, Waltham, MA) was used to generate an RNA transcript. The transcript was cleaned up using the Qiagen RNeasy Mini Kit (Qiagen, Hilden, Germany), run on a 1% agarose gel to confirm the size, and then quantified by multiple readings on a Nanodrop. The concentration and copy number were calculated from the OD readings. Standard curves were generated by freshly diluting transcripts tenfold from 4.0 × 10^6^ to 4.0 copies/µL (2.0 × 10^7^ to 20.0 copies/reaction) before each run. Standard curves were shown to have an average efficiency of 100% based on the slope of the curves. A positive control of extracted A/California/04/2009 H1N1 virus was run in the reaction over 20 times by two technicians over a 5-week period to obtain data to set an acceptance range based on 2 standard deviations of the average quantity obtained in the assay.

To confirm specificity towards A/California H1N1, eight different influenza A and B viruses (Additional file 2: Table S2) were tested in the assay. Only A/California H1N1 specific isolates were detected in the assay. Additionally, to demonstrate the specificity of the primers and probe, the PCR product of the positive virus control was run on a 2% agarose gel and assessed for a band at 177 bp to confirm that only the targeted portion of the gene was amplified (Additional file 3: Figure S1). Intra- assay precision and intermediate precision was determined to have a coefficient of variance (CV) of 11% and 25%, respectively.

The limit of detection (LOD) for the assay was determined from running the standard reference in two-fold dilutions surrounding the lower end of the standard curve in replicates of 20. The LOD was then calculated as the concentration where 95% of the reference standard dilutions gave a positive response (Ct ≤ 40). The LOD was calculated to be 16 copies/reaction. For purposes of comparison with BioFire or rapid test, qPCR samples above the LOD were considered positive, samples below the LOD were considered to be negative.

### BioFire FilmArray

NP swab samples were loaded into the Biofire FilmArray respiratory panel cassette according to the manufacturer’s instructions and analyzed using the BioFire FilmArray Multiplex PCR System (BioMerieux, Marcy-l'Étoile, France) [14]. This device uses a fully automated procedure for nucleic acid purification, amplification, multiplexed PCR, and melting analysis, and generates a report with binary outcomes for various respiratory pathogens. Within assay cassettes are two positive quality controls, the first is an RNA process control to verify successful extraction and reverse transcription, the second is an independent DNA control to verify a successful PCR reaction occurred [14]. All samples analyzed in this study passed both quality controls, and those that were positive for influenza A were considered positive in this report. Samples that were positive for targets other than influenza A were excluded from analysis while all other samples were considered negative.

### Rapid influenza test

NP swab samples were applied to the QuickVue Influenza A + B Test (Quidel, San Diego, CA) using the manufacturer’s instructions [15]. Colorimetric tests were read by eye to determine positive or negative results as per protocol in the test kit insert. All samples reported in this analysis properly displayed positive procedural control lines, indicating successful execution of the test kit protocol [15].

### Statistical analysis

Sensitivity (true positive rate) was calculated as the ratio of true positive results divided by the sum of true positive and false negative results [(true positive)/(true positive + false negative)]. The specificity (true negative rate) was calculated by dividing the number of true negative results by the total number of true negative plus false-positive results [(true negative)/(true negative + false positive)]. The positive and negative predictive values were calculated as true positive divided by all positive results and true negative divided by all negative results, respectively. The confidence interval was calculated using the epiR package [16]. Differences in qPCR copy number values between samples determined to be true positive and false negative by BioFire or rapid test were calculated using the Wilcoxon signed-rank test on log 10 values.

## Results

During a recent volunteer influenza challenge study, 143 healthy volunteers were challenged intranasally with A/California/2009/H1N1 virus [12]. Nasopharyngeal (NP) swabs were routinely collected and tested for the presence of influenza virus as part of the clinical conduct of this study. Multiple NP swabs were collected and used for analysis with either the BioMerieux BioFire FilmArray RP (BioFire) or Quidel QuickVue Influenza A + B Test (rapid test) in addition to the validated qPCR assay, thus providing an opportunity to compare the relative performance of these tests.

To facilitate the analysis described here, the gold-standard qPCR assay was considered to be diagnostically accurate for the detection of influenza A virus in all samples. All values above the LOD by qPCR were recorded as positive, and all samples below the LOD were recorded as negative.

**Fig. 1:**
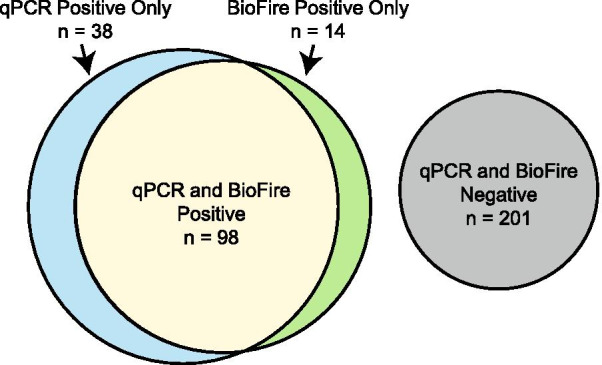
Venn diagram of BioFire performance versus qPCR. Number of samples positive by qPCR (blue), positive by BioFire (green), or positive by both qPCR and BioFire (yellow). Samples negative for both qPCR and BioFire (grey). Size of circles is proportional to n

351 matched NP swab samples (702 total) were tested in parallel using BioFire and qPCR. Of the 136 qPCR positive samples, BioFire correctly identified 98 of the matching samples as positive for influenza A (true positive) and 38 samples as negative for virus (false negative). Of the 215 samples that were qPCR negative for influenza A, The BioFire assay accurately classified 201 samples as negative for virus (true negative) while incorrectly classifying 14 samples as positive (false positive) (Table 1, Fig. 1). As such, the sensitivity and specificity of the BioFire assay relative to qPCR was 72.1% [63.7–79.4%, 95% confidence interval (CI)] and 93.5% (89.3–96.4%, 95% CI), respectively (Table 2). For BioFire, true positive (TP) samples had slightly higher mean qPCR copy number values than false-negative samples (FN) (TP, 5.63 ± 1.07 vs. FN, 5.19 ± 1.14 [mean, ± SD (log10 copies/mL)], *p* = 0.023 [Wilcoxon]). The lowest recorded qPCR copy number associated with a matched BioFire TP sample was 3.74 (log10 copies/mL).

**Table 1:** BioFire versus qPCR and rapid test versus qPCR outcomes

	qPCR positive	qPCR negative
BioFire (n = 351)		
Positive	98	14
Negative	38	201
Rapid test (n = 351)		
Positive	15	1
Negative	161	122

Results for matched tests performed by BioFire and qPCR or rapid test and qPCR

**Table 2:** Sensitivity and specificity of BioFire and rapid test versus qPCR

Diagnostic test	Sensitivity			Specificity		
	TP/(TP + FN)	%	95% CI	TN/(TN + FP)	%	95% CI
BioFire	98/136	72.1	63.7–79.4	201/215	93.5	89.3–96.4
Rapid test	15/176	8.5	4.8–13.7	122/123	99.2	95.6–100

*TPR* true positive rate, *TNR* true negative rate, *TP* true positive, *TN* true negative, *FP* false positive, *FN* false negative, *CI* confidence interval

**Fig. 2:**
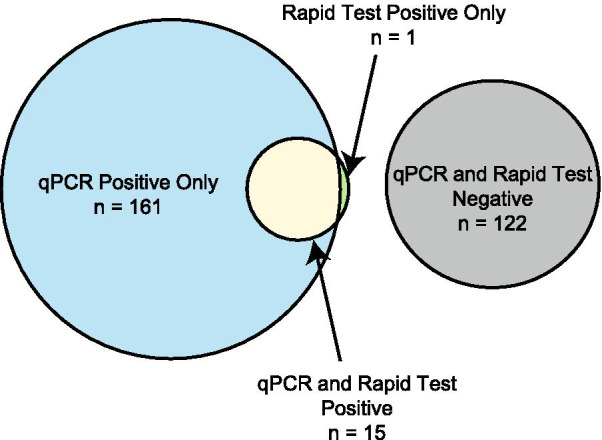
Venn diagram of rapid test performance versus qPCR. Number of samples positive by qPCR (blue), positive by rapid test (green), or positive by both qPCR and rapid test (yellow). Samples negative for both qPCR and rapid test (grey). Size of circles is proportional to n

A similar analysis was completed for 299 matched NP swab samples (598 total) that were tested by both qPCR and rapid test. Of 176 qPCR positive samples, rapid test recorded 15 true positives and 161 false negatives for matching samples. Of the 123 qPCR negative samples, rapid test identified 122 true negatives and one false positive (Table 1, Fig. 2). This resulted in a sensitivity of 8.5% (4.8–13.7%, 95% CI) and specificity of 99.2% (95.6–100%, 95% CI) for rapid test relative to qPCR (Table 2). For rapid test, TP samples had significantly higher mean qPCR copy number values than FN samples (TP, 6.82 ± 1.37 vs. FN, 5.69 ± 1.11 [mean, ± SD (log10 copies/mL)], *p* = 0.003 [Wilcoxon]). The lowest recorded qPCR copy number associated with a matched rapid test TP sample was 4.45 (log10 copies/mL).

Sensitivity, or true positive rate (TPR), was calculated as the number of TP results divided by the sum of TP and FN results. Specificity, or true negative rate (TNR), was calculated as the number of true negatives (TN) divided by the sum of TN and false positives (FP). The 95% confidence interval was calculated using the epiR package in R.

## Discussion

Rapid and simplified methods for influenza virus detection can be valuable tools, but it is important to understand how these tests compare to other assays in different settings. One important setting is during volunteer influenza challenge studies, where detection of virus shedding may be used as a trigger for treatment or other protocol steps. Here the performance of BioFire, a simplified sample-to-answer qPCR-based detection method, and QuickVue Influenza A + B Test rapid test, a colorimetric immunoassay, were assessed by comparing their diagnostic results to a validated qPCR assay using matched samples from an A/California/2009/H1N1 volunteer challenge study. Based on this analysis, BioFire was largely consistent with qPCR, while the rapid test suffered from a large degree of false negatives.

BioFire is an arrayed multiplexed qPCR-based device delivering binary results (positive or negative) for targets included in its assay cassettes. Although BioFire had a high specificity of 93.5%, sensitivity of BioFire was lower at 72.1%. This lower sensitivity may reflect a higher LOD by the BioFire assay for the A/California/2009/H1N1 influenza challenge strain compared to qPCR in the context of this study. A higher LOD could result from differences in sample prep, primer sets, instrumentation, or programmed reporting threshold. Our calculated sensitivity of 72.1% for BioFire was only slightly lower than the 73–89% sensitivity range reported in other studies [17–19]. Taken together, while such assays are simpler to use and offer a quicker turnaround compared to standard qPCR, this may come at the expense of slightly decreased sensitivity.

Rapid influenza diagnostic tests are well known to have decreased sensitivity compared to qPCR [7]. While some studies have reported sensitivities as high as 63–71% [20–22], others report much lower values in the range of 26–33% [23, 24]. In our analysis, the rapid test sensitivity was very low, at only 8.5% relative to qPCR. This wide range of sensitivities reported across studies may be attributed to differences in virus strain or magnitude of antigen load, at least in part, by the day post-infection in which samples are collected. Additionally, as a colorimetric test, the qualitative results are susceptible to greater variation due to biases in operator readings compared to the quantitative driven results of qPCR and BioFire. Nevertheless, the specificity of the rapid test is very high. Our study found a specificity of 99.2% for this test, in line with other reports of 96–100% specificity [20, 21, 23, 24]. Therefore, despite poor sensitivity, a positive rapid test, when present, is a strong indicator that a sample will also be positive by qPCR.

For operational reasons, this study was limited to examining samples starting at 4 days post-viral challenge, corresponding to approximately three days post-symptom onset in challenge models [8]. It is important to understand that cohorts of community-acquired infection self-identifying as symptomatic are fundamentally different from those of volunteer influenza challenge studies, and that the results of this study may not be extensible to community-acquired infection settings. For example, in other studies that examine the performance of influenza detection assays during community-acquired infection [17, 20, 21, 23, 24], NP samples were collected immediately when patients met a set of subjective symptom criteria. Symptom-based self-selection may bias for the inclusion of individuals in those studies with higher magnitudes of viral titer and antigen load compared to a controlled challenge study in which all study participants are automatically assessed at predetermined time points. These differences may explain the reduced overall performance of the rapid test in this study compared to prior studies. The requirement of higher viral loads to trigger a positive rapid test in this study is suggested by significantly higher qPCR copy numbers for rapid test TP samples versus rapid test FN samples, an effect that was less pronounced for BioFire. Nevertheless, the data reported here is important for our broader understanding of the performance of these diagnostic tools, especially for those considering their use in future influenza challenge studies. If available from future influenza challenge studies, an examination of samples taken over the entire course of virus shedding period, including from earlier time points post-inoculation would be worthwhile to determine the relationship between day post-infection, viral load, and performance of both rapid tests and sample-to-answer molecular tests for influenza A.

## Conclusion

In summary, we have compared the performance of two simplified influenza A detection methods to a validated qPCR assay in a controlled A/California/2009/H1N1 challenge study. In this setting, BioFire closely reflected virus shedding detected by qPCR, while the rapid test did not. While not without limitations, integration of sample-to-answer influenza tests such as BioFire into environments where standard qPCR assays are impractical stands to greatly improve detection of influenza over antigen-based rapid tests alone.

## Supplementary Information

**Additional file 1: Table S1**. qPCR Primers and probes. Sequences of the qPCR primers and probes used to target the HA gene of the pandemic (pdmH1) influenza A (H1N1) 2009 virus.

**Additional file 2: Table S2**. Confirmation of Specificity of qPCR test for A/California H1N1. Eight different influenza A and B strains were used to test the specificity of the qPCR assay. Ct values confirm assay is specific for A/California H1N1 viruses.

**Additional file 3: Figure S1**. Confirmatory PCR product from positive virus control. An expected single band of ~177bp amplified by PCR demonstrates the specificity of the primers to amplify the targeted region of the HA gene.

## Data Availability

Information about the intranasal influenza A/California/2009/H1N1 challenge can be found at ClinicalTrials.gov Identifier: NCT02918006. Other datasets used and/or analyzed during the current study are available from the corresponding author on reasonable request.

## Abbreviations

CDC: US Centers for Disease Control and Prevention
CI: Confidence interval
CV: Coefficient of variance
LOD: Limit of detection
NP: Nasopharyngeal
pdmH1: Pandemic influenza A (H1N1) 2009
qPCR: Quantitative real-time polymerase chain reaction
TPR: True positive rate
TNR: True negative rate
TP: True positive
TN: True negative
FP: False positive
FN: False negative
