# Correction to: Performance of BioFire array or QuickVue influenza A + B test versus a validation qPCR assay for detection of influenza A during a volunteer A/California/2009/H1N1 challenge study

**DOI:** 10.1186/s12985-021-01531-1

**Published:** 2021-03-15

**Authors:** David R. McIlwain, Han Chen, Maria Apkarian, Melton Affrime, Bonnie Bock, Kenneth Kim, Nilanjan Mukherjee, Garry P. Nolan, Monica M. McNeal

**Affiliations:** 1 Department of Pathology, Stanford University School of Medicine, Stanford, CA USA; 2 WCCT Global, Cypress, CA USA; 3 ARK Clinical Research, Long Beach, CA USA; 4 Department of Pediatrics, Division of Infectious Diseases, University of Cincinnati College of Medicine, Cincinnati Children’s Hospital Medical Center, Cincinnati, OH USA

## Correction to: Virol J (2021) 18:45 https://doi.org/10.1186/s12985-021-01516-0

Following publication of the original article [1], the author notified us that there is an error in Table 1, originated during typesetting. The correct Table 1 is given below.

**Table 1:** BioFire versus qPCR and rapid test versus qPCR outcomes

	qPCR positive	qPCR negative
BioFire (n = 351)		
Positive	98	14
Negative	38	201
Rapid test (n = 299)		
Positive	15	1
Negative	161	122

Results for matched tests performed by BioFire and qPCR or rapid test and qPCR.

The publisher apologizes for any inconvenience.

The original article has been corrected.
