# Supplementary figures and images for: Performance of BioFire array or QuickVue influenza A + B test versus a validation qPCR assay for detection of influenza A during a volunteer A/California/2009/H1N1 challenge study
Source: Virol J. 2021 Feb 25;18:45. doi: 10.1186/s12985-021-01516-0 (PMC7905982; doi:10.1186/s12985-021-01516-0)

Figure S1

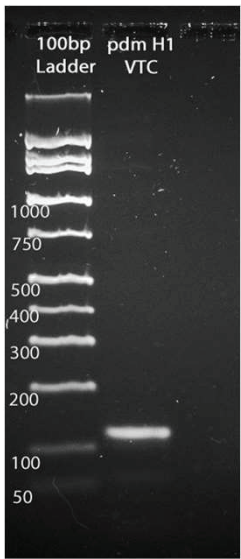

Supplement: Supplementary file 3 — Additional file 3: Figure S1. Confirmatory PCR product from positive virus control. An expected single band of ~177bp amplified by PCR demonstrates the specificity of the primers to amplify the targeted region of the HA gene. [file 12985_2021_1516_MOESM3_ESM.pdf]
